# Supplementary material for: Saccades are locked to the phase of alpha oscillations during natural reading
Source: PLoS Biol. 2023 Jan 17;21(1):e3001968. doi: 10.1371/journal.pbio.3001968 (PMC9882905; doi:10.1371/journal.pbio.3001968)
Supplement: S1 Text — (DOCX) [file pbio.3001968.s002.docx]

**S1 Text. Two behavioural pre-tests of the sentences**

*Pre-test for the plausibility of the whole sentence*

We created a 7-point rating scale to test the plausibility of sentences. Because the sentences in this study were supposed to be highly plausible (e.g., sentence 2 in the table below), we constructed another 142 filler sentences to occupy the full range of the scale. Half of the filter sentences were of middle plausibility (e.g., sentence 1), and the other half were of very low plausibility (e.g., sentence 3).

|  | Implausible Plausible  1 2 3 4 5 6 7 |
| --- | --- |
| 1. Kate said that she saw lots of stars twinkling in the sky at noon. | 1 2 3 4 5 6 7 |
| 2. It was obvious that the beautiful music captured her attention. | 1 2 3 4 5 6 7 |
| 3. Sarah used an adhesive to glue the traffic and just made it to the wedding. | 1 2 3 4 5 6 7 |
| …… | 1 2 3 4 5 6 7 |

We asked participants to rate the plausibility (or acceptance) of each sentence. For version A of the first sentence set, 22 participants were recruited (20 females), but 2 participants were excluded due to incomplete or careless responses. The plausibility rating for the sentences used in this study was 5.7 ± 0.5 (mean ± SD), significantly higher than the rating for the filler sentences (*t*_(19)_ = 23.98, *p* < 0.001, pairwise t-test, two-tailed). The same result was found for version B from 23 participants (19 females). The plausibility rating for experimental sentences (5.5 ± 0.8, mean ± SD) was significantly higher than the filler sentences (*t*_(22)_ = 16.68, *p* < 0.001, pairwise t-test, two-tailed). The pre-test indicated that the sentences used in the current study were highly plausible.

*Pre-test for the predictability of target words*

We conducted a cloze pre-test to test the predictability of the target words. A sentence fragment that up to but did not include the target word was shown (see the example below). And participants were instructed to write down the first word/s that come to mind in order to complete the sentence.

Mike thought this difficult _________________

We defined the target word in a given sentence as unpredictable when this word was predicted by less than 10% of the participants. For the first round of the cloze test, 22 participants (21 females) were recruited, and 6 target words turned out to be predictable. Then we replaced these target words and carried out another round of cloze test. Twenty-two new participants (19 females) were recruited, and no target words were predicted by more than 2 participants. The predictability cloze test made sure that all the target words were not predictable from the sentence context.
