# Supplementary material for: Saccades are locked to the phase of alpha oscillations during natural reading
Source: PLoS Biol. 2023 Jan 17;21(1):e3001968. doi: 10.1371/journal.pbio.3001968 (PMC9882905; doi:10.1371/journal.pbio.3001968)
Supplement: S1 Table — Note. Position refers to the word location in a sentence where the pre-target, target, or post-target words is presented, with the unit of words. Word length means the number of letters in a given word, with the unit of letters. Lexical frequency is measured in terms of total CELEX frequency per million, where the frequency is lower than 10 for the low lexical frequency target words and higher than 30 for the high lexical frequency target words. All values in the table are mean values with standard deviations in the parentheses (mean ± SD). (DOCX) [file pbio.3001968.s003.docx]

**S1 Table. Characteristics of words used in the current study**

Pre-target Low frequency target High frequency target Post-target

Lexical frequency 359.9 (1109.3) 5.3 (4.5) 95.3 (135.5) 569.3 (1734.7)

Word length 6.1 (1.5) 5.8 (0.8) 5.8 (0.8) 6.7 (1.7)

Position 5.7 (2.3) 6.7 (2.3) 6.7 (2.3) 7.7 (2.3)
