# Supplementary material for: Saccades are locked to the phase of alpha oscillations during natural reading
Source: PLoS Biol. 2023 Jan 17;21(1):e3001968. doi: 10.1371/journal.pbio.3001968 (PMC9882905; doi:10.1371/journal.pbio.3001968)
Supplement: Attachment — Submitted filename: Response to Reviewers.pdf [file pbio.3001968.s011.pdf]

Editor: We will be particularly looking to see you respond with further analyses to two of the major issues raised (i.e. Reviewer 1: The actual phase of alpha for the different word frequency conditions;

We did some further analyses to address this (see S4 Fig). The conclusion is that the absolute alpha phase was different across participants. This is a typical observation in other studies as well because the underlying cortical generators of alpha activity exhibit a different orientation with respect to the MEG sensors across participants. We also tested if there was a different alpha phase when saccading to low versus high-frequency words. This was not the case (see details below).

Reviewer 2: possible effects in other MEG frequency bands.

We now extended the analysis to other frequency bands (see S5 Fig). The observation of saccades locking was constrained to the phase of the alpha band (see detail below).

While recognizing that directly demonstrating causality could prove difficult, please note that we would also be looking to see whether your revisions convince these reviewers that the overall findings now provide the extent of conceptual advance suitable for PLOS Biology.

We have now downplayed some of the statements that might have been interpreted as causal arguments. We now make explicit that the link between the alpha phase and saccades is correlational. We also point to how to test a causal link using TMS to the frontal eye fields (see the response to Reviewer 1).

Reviewer #1: This short manuscript reports effect of stronger phase consistency of alpha-band oscillations during natural (sentence) reading, at the onset of saccades that precede target words of great lexical complexity or lesser frequency. This report is aligned with a vein of interesting and relevant literature on the role of human alpha oscillations for modulating the inhibition of cortical circuits and/or gating the flow of information processing across brain networks. Overall, the manuscript is engaging and clearly written, with clear and relevant illustrations. However, the depth of the findings remain relatively anecdotal and may not be of sufficient interest to the broad readership of the journal.

We thank the Reviewer for these critical comments and for their constructive questions that helped us further improve the manuscript. Given the strong interest in brain oscillations and several recent papers linking (micro-)saccades to alpha oscillations [Baiwei Liu, et.al., 2022, bioRxiv; Baiwei Liu, et.al., 2022, NC] we do believe that our findings will be of broad interest. We would like to stress that there is a shift in the field in which alpha oscillations typically were considered a 'visual rhythm'; there is now increasing evidence that alpha oscillations are important for visuomotor coordination (Tzvetan Popov, et.al., bioRxiv, 2021) and our paper is a novel contribution speaking to the phasic role of alpha oscillations.

References:

Liu, Baiwei, Anna C. Nobre, and Freek van Ede. "Microsaccades transiently lateralise EEG alpha activity." bioRxiv (2022).

Liu, Baiwei, Anna C. Nobre, and Freek van Ede. "Functional but not obligatory link between microsaccades and neural modulation by covert spatial attention." Nature communications 13.1 (2022): 1-10.

Popov, Tzvetan, et al. "Alpha oscillations link action to cognition: An oculomotor account of the brain's dominant rhythm." bioRxiv (2021).

In my opinion, one major shortcoming of the report is that it frames the study and the discussion of the results as if the data established a causal role of phase effects of alpha oscillations on reading behavior in general, and in supporting lexical processing and/or the coordination between the visual and oculomoteur systems. Such causal demonstration is not derived in the presented work. The manuscript simply reports an association, arguably interesting in the sense that it is in line with previous observations, between the consistency of the phase of alpha oscillations across trials prior to saccading towards words and their lexical frequency.

We thank the Reviewer for this comment, which prompted us to revise the discussion section to reduce the causal explanation of the study and emphasise more on the association role. We have now added some discussion on this point in the updated manuscript with tracked changes on page 14 (line 263-270):

“However, please note that the modulation of alpha phase locking before saccades is based on an association analysis rather than a causal analysis. Further studies that involve causal manipulation, such as brain stimulation, are needed to establish a more causal role of phase effects of alpha oscillations on the processing of lexical information or reading behaviour in general. In the past, it has been shown that theta burst TMS to the frontal eye fields (FEF) impair the ability to modulate alpha oscillations by spatial attention [41]. A similar approach could be applied to causally establish the role of alpha band oscillations for visuomotor coordination during reading.”

References:

41. Marshall TR, O’shea J, Jensen O, Bergmann TO, O’shea J, Bergmann TO. Frontal Eye Fields Control Attentional Modulation of Alpha and Gamma Oscillations in Contralateral Occipitoparietal Cortex. J Neurosci. 2015;35: 1638–1647. doi:10.1523/JNEUROSCI.3116-14.2015

The effects are reported in terms of phase-locking across trials and compared between the two conditions of the experiment (frequent vs. less frequent words). It would be much more significant to show whether the actual phase values of alpha oscillations are different between conditions, in addition to reporting the alignment of said phase across trials. Indeed, the authors subscribe to the notion that the alpha oscillatory cycle marks time segments of lesser to stronger inhibition of cortical circuits and that stronger phase consistently between trials prior to saccading to more challenging words is a marker of the contextual modulation of such inhibitory mechanisms. However, without also exploring and reporting the consistency of the actual phase value, the paper does not entirely respond to its conceptual framework and our expectations. For instance, if less inhibition of certain visual circuits is required to process more challenging words, this should manifest with a greater aggregation of phase angle values around a certain phase segment of the alpha cycle, and the mode of such aggregation should be different between experimental conditions.

We agree with the Reviewer that the actual alpha phase value is of great importance for the current study. We analysed the preferred alpha phase during the pre-target intervals for each condition and reported this result in the revised manuscript (page 10, line 188-196 with tracked changes):

“Although the consistency of alpha phase over trials was stronger when previewing a low lexical frequency word compared with a high lexical frequency word, the actual preferred alpha phases for each condition are not consistent across participants (S4 Fig, B). This is likely explained by the fact that the underlying cortical generators of alpha activity exhibit a different orientation with respect to the MEG sensors across participants. The resulting variability in dipole orientation will also result in different absolute phases of the alpha oscillations in the MEG data. In addition, we did not find a consistent phase difference in the alpha band with respect to previewing low and high frequency words (see S4 Fig, F).”

We also added the result figure into the supplementary material on page 40 with tracked changes:

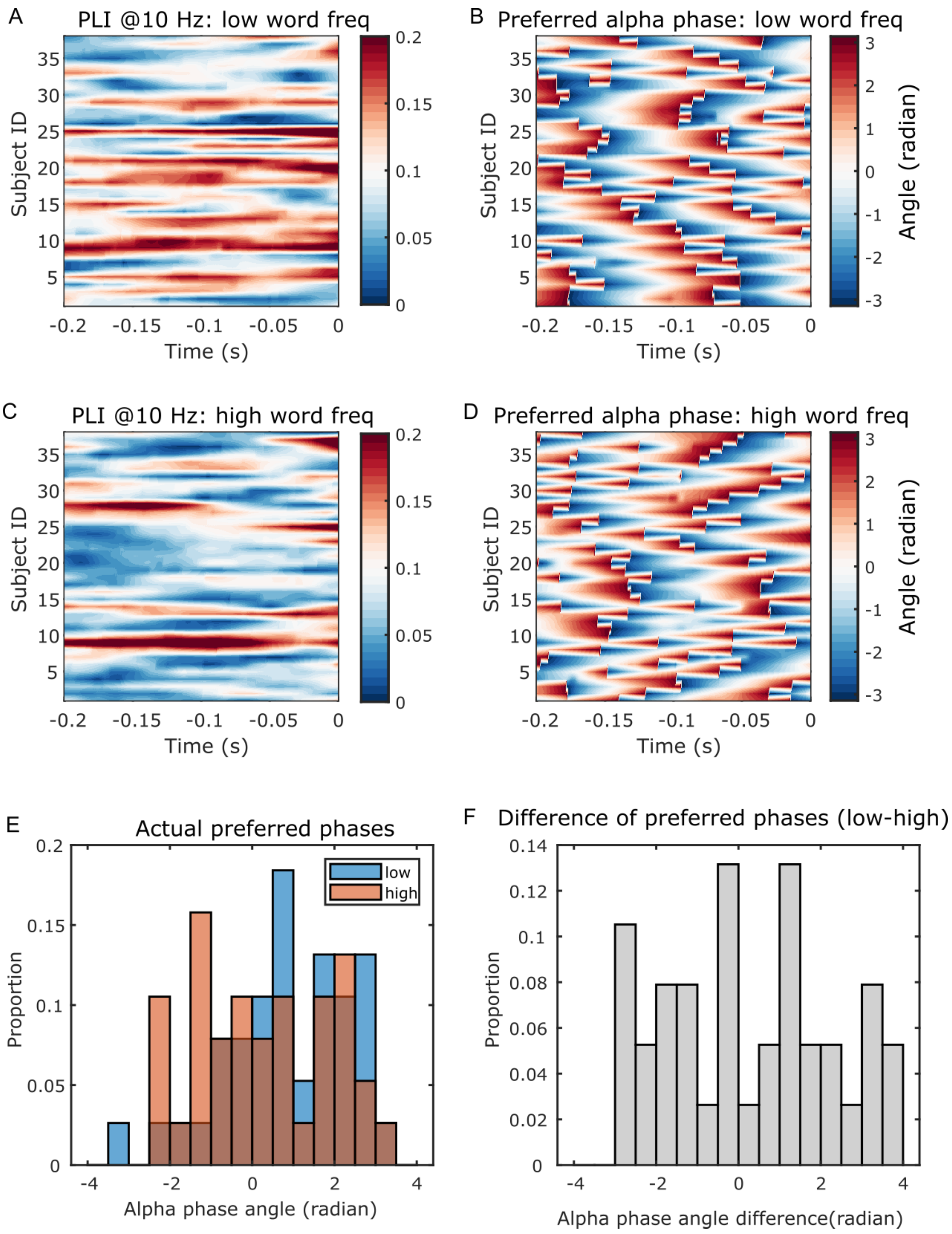

*"**S4 Fig. The absolute preferred alpha phase during the pre-target intervals.** We selected the planar sensor that showed the strongest phase-locking difference between conditions in Fig 3 (B) and plotted the phase locking index (PLI) at 10 Hz before saccade onset (zero time point) to the target words with low word frequency (A) and high word frequency (C). The preferred alpha phase angles over all participants (n = 38) under the low and high target condition were shown in (B) and (D) separately. (E) The distribution of the preferred alpha phase angles at -0.1 s over participants for low (in blue) and high (in orange) condition. (F) Distribution of difference of the preferred alpha angle between conditions was not significantly different from a uniform distribution (Kolmogorov-Smirnov test, p = 0.34)"*

Another issue with the results presented is with the significance of the brain region shown to demonstrate greater phase consistency in the "challenging-word" condition: MEG source mapping

localizes to the dorsal aspect of the parietal region, which functional significance is uncertain. For instance, how does this region relate to oculomotor control? How is it related to parafoveal visual/reading processes that are essential in such task, in order to realign the phase of alpha oscillations. The proposed Discussion only scratches the surface of these important questions and leaves the reader with an impression that the study is incomplete and has missed opportunities to go deeper into key systems neuroscience aspects that are arguably complex, but within reach of the data.

We thank the Reviewer for this comment as it helped us clarify the significance of the source localization result that was not sufficiently elaborated on in the original version of our manuscript. We added more discussion on this point in the revised manuscript (on page 15, line 272-286 with tracked changes):

"This result indicates that the phase modulation originated from the dorsal part of the parietal region, which is of great functional significance to both oculomotor control and natural reading. First, the parietal cortex is part of the oculomotor control network that involves the frontal eye fields, motor, and visual cortex [42]. Nonhuman primates' studies found that the lateral intraparietal (LIP) area codes the motor coordinates of saccades [43] and is involved in decisions about when to initiate the saccade and where to look, as well as where not to look [44]. Second, the dorsal stream of the parietal cortex is believed to be implicated in visuospatial attention [45] and damage in the posterior parietal cortex results in the core deficit of dyslexia [46]. Third, human electrophysiological studies found that alpha oscillations play a functional role within the oculomotor control network [41,47], linking oculomotor action to cognition [15]. Therefore, in the current study, alpha oscillation from the dorsal pathway might serve to coordinate the communication within the oculomotor control network, which is modulated by the previewed information during reading."

References:

15. Popov T, Miller GA, Rockstroh B, Jensen O, Langer N. Alpha oscillations link action to cognition: An oculomotor account of the brain's dominant rhythm. bioRxiv. 2021. doi:10.1101/2021.09.24.461634

41. Marshall TR, O'shea J, Jensen O, Bergmann TO, O'shea J, Bergmann TO. Frontal Eye Fields Control Attentional Modulation of Alpha and Gamma Oscillations in Contralateral Occipitoparietal Cortex. J Neurosci. 2015;35: 1638–1647. doi:10.1523/JNEUROSCI.3116-14.2015

42. Martinez-Conde S, Otero-Millan J, MacKnik SL. The impact of microsaccades on vision: towards a unified theory of saccadic function. Nat Rev Neurosci 2013 142. 2013;14: 83–96. doi:10.1038/nrn3405

43. Barash S, Bracewell RM, Fogassi L, Gnadt JW, Andersen RA. Saccade-Related Activity in the Lateral Intraparietal Area II. Spatial Properties. J Neurophysiol. 1991;66: 1109–1124.

44. Schiller PH, Tehovnik EJ. Look and see: how the brain moves your eyes about. Prog Brain Res. 2001;134: 127–142. doi:10.1016/S0079-6123(01)34010-4

45. Saalmann YB, Pigarev IN, Vidyasagar TR. Neural mechanisms of visual attention: How top-down

feedback highlights relevant locations. Science (80-). 2007;316: 1612–1615. doi:10.1126/SCIENCE.1139140

46. Vidyasagar TR, Pammer K. Dyslexia: a deficit in visuo-spatial attention, not in phonological processing. Trends Cogn Sci. 2010;14: 57–63. doi:10.1016/j.tics.2009.12.003

47. Popov T, Kastner S, Jensen O. FEF-Controlled Alpha Delay Activity Precedes Stimulus-Induced Gamma-Band Activity in Visual Cortex. J Neurosci. 2017;37: 4117–4127. doi:10.1523/JNEUROSCI.3015-16.2017

In addition to these major issues, I have technical questions for the authors to clarify concerning MEG source mapping. The main text introduces the MEG source maps as produced from the PLI sensor values (which would be a biophysically invalid thing to do because PLI measures are statistics, not physical values). However, the Methods section mentions that the PLI values were actually derived at the source level (which is correct). Yet, these latter were obtained from projecting the Hilbert transformed sensor data into source space using an LCMV beamformer. The authors need to justify the physical validity of using the LCMV kernel, designed to project magnetic field sensor data, with the complex values of the analytical signal, which are physically meaningless. For instance, the LCMV kernel includes data covariance statistics, and I wonder how these latter differ from those of the analytical signals.

We thank the Reviewer for this comment, which prompted us to explain our rationale for source localization in more detail. As the Reviewer pointed out LCMV kernel is not suitable to be applied to the analytical signal. In our study, we did not apply LCMV kernel to the complex values of the analytic signal but applied the weights matrix from the LCMV kernel to the complex values of the analytic signal. We use the same method as described in this paper (Bardouille, T., & Ross, B. (2008). MEG imaging of sensorimotor areas using inter-trial coherence in vibrotactile steady-state responses. Neuroimage, 42(1), 323-331.) and a thread from the Fieldtrip email list (https://mailman.science.ru.nl/pipermail/fieldtrip/2011-March/016482.html). We apologize for the confusion and have now rewritten the procedure of source localization in the revised manuscripts (with tracked changes in the following pages).

Main text (on page 13, line 230-232): "We used a beamformer technique, Linearly Constrained Minimum Variance (LCMV) [33] to calculate the PLI in the source space in order to localize the generators that produced the PLI initially observed in the sensors."

Methods section(on page 28, line 603-614): "Next, in the sensor space, we applied the Hilbert transformation to the 9 – 13 Hz filtered data to estimate the signals in the alpha band for each trial. The weight matrix from the common LCMV filters were applied to these bandpass filtered signals in the -0.2 – 0 s interval to estimate the signals in the source space (for methodological details please see [73]). Finally, we applied Equation (2) to the source space data to estimate the PLI time series in the source space, which were then averaged across time to obtain the average PLI value for each condition."

References:

73. Bardouille T, Ross B. MEG imaging of sensorimotor areas using inter-trial coherence in vibrotactile steady-state responses. Neuroimage. 2008;42: 323–331. doi:10.1016/j.neuroimage.2008.04.176

Also, the Methods section mentions ITC (acronym undefined but I suppose it stands for inter-trial coherence) for deriving source PLIs, which is inconsistent.

We thank the Reviewer for pointing out this typo and apologize any confusion caused. This typo has been fixed in the revised manuscript (on page 27, line 571 with tracked changes).

Finally, the derivation of data covariance estimates over short periods of time (200 ms) is numerically challenging (especially for alpha oscillations which cycle is 100 ms long, typically) and more detail are required beyond mentioning regularization.

We thank the Reviewer for this great suggestion, which we followed and made us update the source localization result in Figure 3 in the main text (on page 12 with tracked changes in the updated manuscript):

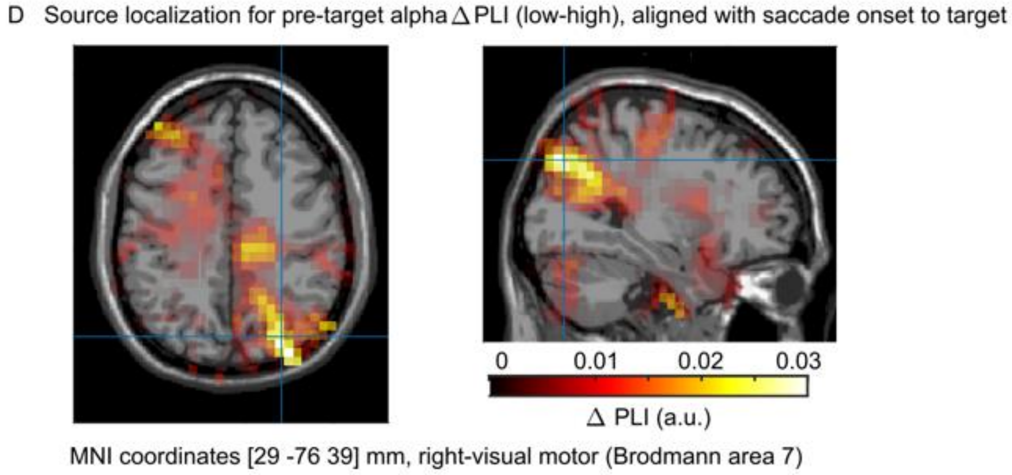

We revised the Methods about Figure 3 on page 28, line 593-597 with tracked changes:

"……the covariance matrix was calculated for the -0.5 to 0 s interval of the filtered alpha band data over all trials regardless of conditions (please note that the estimation of covariance matrix over short periods of time is numerically challenging, so here we selected a longer time window than -0.2 to 0 s, although both time windows gave the nearly identical result, see S7 Fig)."

We also put the old result of source localization into the supplementary figures on page 43 with tracked changes:

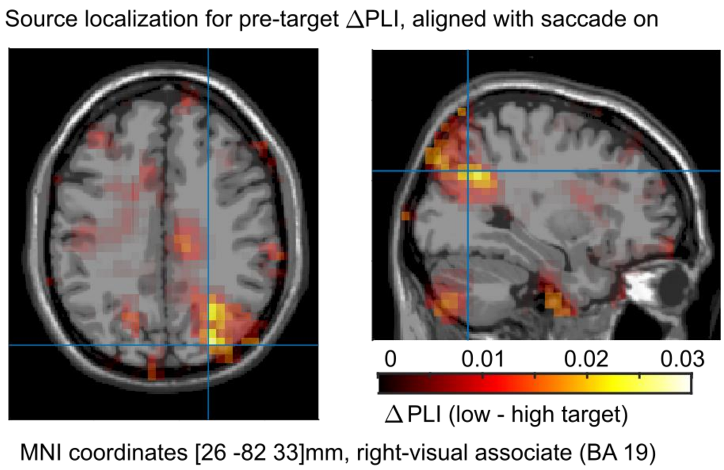

*"**S7 Fig. Source localization of the PLI effect with a covariance estimation time window of -0.2 to 0 s.** The covariance matrix in the alpha frequency band (9 – 13 Hz) was derived from the 0.2 s time window where the PLI difference was significant in the sensor space. Please note that the source localization result was nearly identical as in the main text using a -0.5 – 0 s time window. The difference of localized BA areas was within the error of MEG recording"*

Another open question is how PLI values were derived at reach source location as no orientation constraint was used and therefore 3 time series are produced at each cortical location.

We thank the Reviewer for pointing out this important information that was missing in the original manuscript. We now added a description of the orientation selection in the Methods section of the source analysis on page 28, line 598-601 (with tracked changes in the new manuscript):

"…… The common LCMV filters were created using this covariance matrix and the source model. We specified the LCMV filters to have a fixed dipole orientation at each source grid point, which was the optimal orientation that had the most variance. Thus, the source orientations did not vary over time."

Reviewer #2: This study investigated alpha band oscillatory activity during a natural reading task from a previous data set that manipulated the lexical frequency of a target word in a sentence. The results are consistent with a phase reset in alpha around the saccade onset time period. This reset seems stronger when the upcoming target word has low lexical frequency. These effects were localized to visual processing areas.
Overall, the finding is interesting and potentially important.

We thank the Reviewer for these positive and helpful comments and hope that we were able to address the remaining concerns.

Major comment: The authors focus analyses on the 9-13 hz frequency bands. Only 4-30 Hz was considered. This means that any potential effects in the theta (or delta) bands are not considered. Given the long-standing link between saccades and theta this should be considered and explained. There is also the possibility that higher frequency effects (between 13 -30 hz) might be found, in particular in the post-saccadic interval (see Figure 3C). The authors only consider 9-13 Hz (as stated in the Methods, lines 445-446), which gives an incomplete picture given other studies in the literature showing theta or beta band effects with respect to reading and/or eye movements.

We thank the Reviewer for this point and agree that other frequency bands should be considered to give a complete picture of the current study. We did the same analysis as in the alpha frequency band for the delta (1 – 3 Hz) and theta (4 – 8 Hz) bands before saccades onset to the target words, as well as the beta band (13 – 30 Hz) after saccades onset to the target words. We added the results of these new analyses in the main text and the figure in the supplementary material in the revised manuscript with tracked changes.

Main text (page 11, line 201-206): "To test the specificity of the alpha frequency band, we did the same PLI analysis for the delta (1 – 3 Hz), theta (4 – 8 Hz), and beta (13 – 30 Hz) frequency bands. However, no significant difference between the low and high frequency target lexical conditions was observed (S5 Fig). Together, these results indicate the special functional role of alpha oscillations in coordinating the oculomotor and visual system during natural reading."

Supplementary material (page 41):

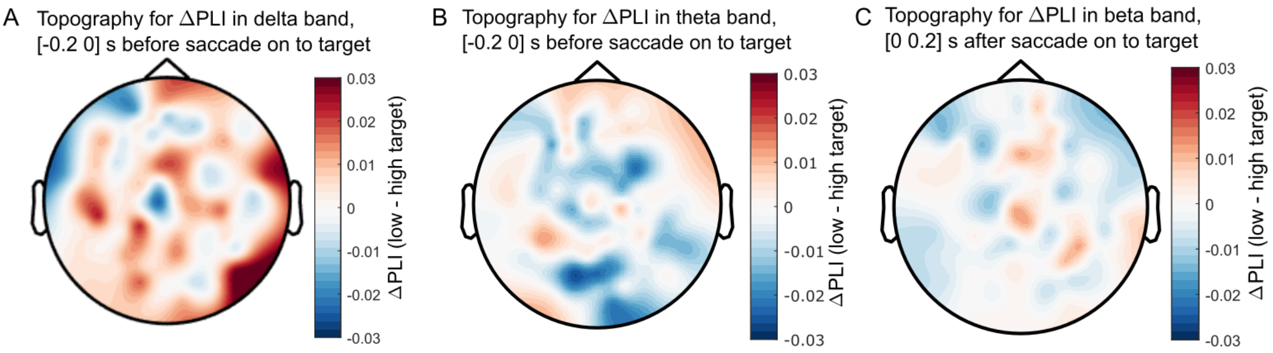

*“**S5 Fig. PLI difference between the low and high target lexical conditions on other frequency bands.** We calculated the PLI values in the delta (1 – 3 Hz) and theta (4 – 8 Hz) frequency bands during -0.2 – 0 s before saccades onset to the target words, as well as in the beta frequency band (13 – 30 Hz) during 0 – 0.2 s after saccades onset to the target words. However, no sensors survived the cluster-based permutation test of the conditional contrast (pairwise, two-tailed t-test).”*

Minor comments

Line 89: Regarding alpha phase and saccades, with behavior, these studies seem relevant (both for the Introduction and even more for the Discussion:

Benedetto & Morrone (2019). Visual sensitivity and bias oscillate phase-locked to saccadic eye movements, Journal of Vision.

Wutz et al (2016). Temporal Integration Windows in Neural Processing and Perception Aligned to Saccadic Eye Movements, Current Biology.

We thank the Reviewer for recommending these interesting papers, which are cited in the revised manuscript with tracked changes.

Introduction section (on page 4, line 94-99): “This pre-stimulus phase in slow frequency oscillations was also found to play an important role in parsing continuous sensory input, determining whether to integrate or segregate two stimuli over time [26]. Behavioural performance of a similar temporal binding task was found to vary with phase locking to saccades, indicating an oscillatory relationship between the saccadic eye movements and the timing of visual processing [27].”

Discussion section (on page 18, line 365-367): “Furthermore, studies that involved eye movements found that saccades had a functional role in …… , and temporal binding [26, 27]”

References:

26. Wutz A, Muschter E, van Koningsbruggen MG, Weisz N, Melcher D. Temporal Integration Windows in Neural Processing and Perception Aligned to Saccadic Eye Movements. Curr Biol. 2016;26: 1659–1668. doi:10.1016/j.cub.2016.04.070

27. Benedetto A, Morrone MC. Visual sensitivity and bias oscillate phase-locked to saccadic eye movements. J Vis. 2019;19: 15–15. doi:10.1167/19.14.15

Line 124: "Consistent with the literature, this suggests that eye-tracking data alone do not provide support for lexical parafoveal processing." - given that the question of what information is gleaned from parafoveal processing has not been raised or at all introduced in the Introduction, this comes from nowhere within the text, is a bit complex in meaning ("eye tracking data ALONE"…), does not cite any literature supporting this statement, and perhaps does not do service to the complexity of this question within the reading literature. Given that perhaps the most interesting finding is the alpha phase locking for low-frequency words, something about parafoveal processing should be introduced earlier in the ms.

We thank the Reviewer for pointing this out as it helps us to give a more complete introduction section. Now we added a brief review of the literature about lexical parafoveal processing in eye movement studies in the revised manuscript with changes tracked.

Introduction section (on page 2, line 47-50): "Although the visual acuity in the parafovea is relatively low, information can still be extracted [2]. Eye movement studies have found robust evidence of parafoveal processing on the sub-lexical level but have difficulties finding it on the lexical level (for a comprehensive review please see [3])."

Result section (on page 8, line 135-141): "Consistent with the literature [3, 29], this suggests that eye-tracking data alone do not provide support for lexical parafoveal processing."

References:

2. Rayner K, Bertera JH. Reading without a fovea. Science (80-). 1979;206: 468–469.

3. Schotter ER, Angele B, Rayner K. Parafoveal processing in reading. Attention, Perception, Psychophys. 2012;74: 5–35. doi:10.3758/s13414-011-0219-2

29. Brothers T, Hoversten LJ, Traxler MJ. Looking back on reading ahead: No evidence for lexical parafoveal-on-foveal effects. J Mem Lang. 2017;96: 9–22. doi:10.1016/j.jml.2017.04.001

Lines 127-134 and Figure 2: It is difficult to interpret the statements about alpha "dominating" or "prevailing" (not sure about either of these choices of wording) without some context. This context is not provided until the end of the Discussion (line 227).

We thank the Reviewer for this comment, we have added some context about alpha oscillations in the revised manuscript (on page 9, line 153-159 with changes tracked).

"Alpha oscillations have long been thought to be depressed (or blocked) when processing visual stimuli [31]; however, this notion stems mainly from paradigms where visual stimuli are presented one by one on the screen. Here we show that alpha oscillations remain strong over parietal and occipital regions when processing words during natural reading. This points to the importance of using naturalistic situations to study the functional role of alpha oscillations."

It is not clear if this includes some baseline or is simply a measure of oscillatory power in general, in which case one should expect 1/F power distributions (not sure if this corrects for that, as might be done with the FOOF toolbox for example). Wouldn't something like 1/F (plus perhaps some suppression of theta) give you the same pattern?

We thank the Review for pointing out the 1/F issue, which helps us to identify more clearly the oscillatory components in the data. We added the FOOOF analysis in the revised manuscript on page 8 (line 145-149 with tracked changes).

"The raw power was first averaged across sensors and then across all participants to obtain the grand average. We also applied the FOOOF analysis [30] to separate the aperiodic component (1/f

distribution) and the oscillatory component from the power spectrum, which showed a clear peak around the alpha frequency band (Fig 2B).”

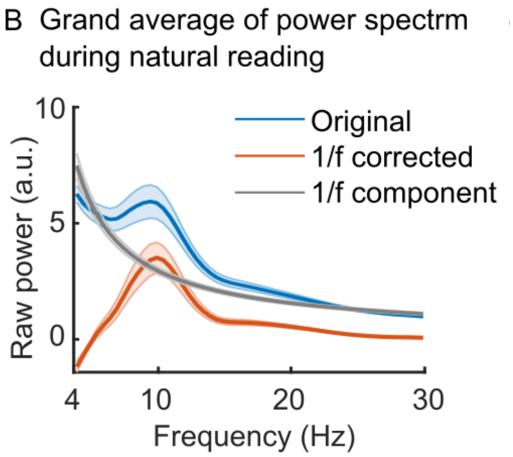

“Fig 2. (B) The grand averaged power spectrum of the original data in (A), which was separated into the 1/f component (in grey) and the 1/f-correct components (in orange). The shaded areas indicate the standard error over participants (n = 38).”

The 8-12 hz band (or 9-13 hz in this ms) is called "alpha" for a reason, in that it is the dominant pattern that can often be seen clearly even in the raw EEG/MEG trace (especially during resting state). By only looking at raw power without any baseline for comparison, it is not clear whether alpha is indeed reduced or increased relative to other conditions. I get the point, but from the analysis, it could theoretically be the case that alpha power is greatly diminished compared to some other condition.

Also, because there is no control condition, such as saccade-only (no reading), we do not know whether the raw alpha power is driven by the saccade, by the reading task, or both.

We thank the Reviewer for this comment and agree that without any baseline correction it’s not clear whether alpha oscillations are reduced or increased during reading. As the Reviewer mentioned, since we don’t have a control condition where participant saccade around without reading, we use the whole time window of the raw power that showed in Fig 2A to correct the raw power. In this way, we can show the power changes during reading with respect to the saccades. From this plot note that the alpha power is strong prior to the saccade are reduced after. As such we can exclude the possibility that alpha is generated by the spectral context in fixation related fields.

We updated this analysis in the result section (page 8, line 144-145 with changes tracked in the revised manuscript):

“(……for the baseline corrected time-frequency representation please see S1 Fig.) ”

We also added the result figure into the supplementary material (on page 37 with tracked changes):

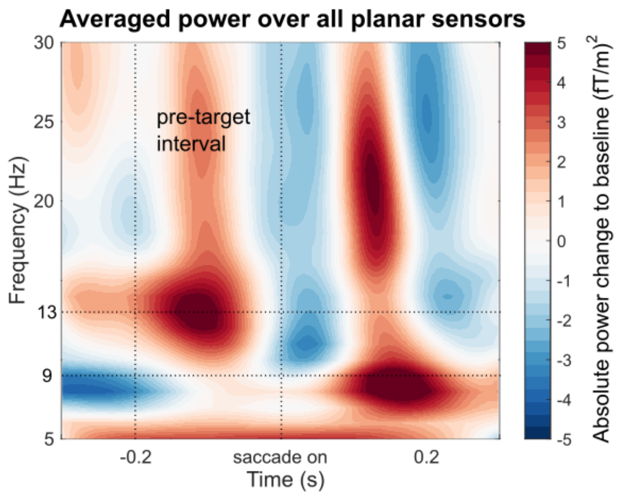

*"**S1 Fig. The corrected time-frequency representation during natural reading**. First, we averaged the power over all trials, all planar sensors, and all participants to obtain the grand average raw power. The "baseline power" was estimated by averaging the raw power over the whole temporal window. We then subtracted this baseline power from the raw power at each frequency. The vertical dashed lines indicate the start of the pre-target interval and the saccade onset (t = 0 s) to the target words."*

In searching for saccades and alpha, this paper looks at alpha power and phase with saccades and no task (which seems quite relevant to this ms):
Acunzo & Melcher. Changes in alpha and theta band activity at the time of saccades: separate contributions of visual and non-visual signals. PsyArXiv. https://doi.org/10.31234/osf.io/c29xm

It would seem relevant for interpreting the effects in this submission, in particular their Figures 2 and 5 showing power and phase coherence effects locked to saccade onset.

In sum, at best this figure in its present form seems to show that alpha is not eliminated during the task, which is not how it is portrayed in the text.

We thank the Review for suggesting such an interesting and highly relevant paper, which we cited to help with the explanation and discussion in the updated manuscripts (on page 17, line 330-335 with tracked changes):

"In a recent MEG study, Acunzo and Melcher distinguished the neural mechanisms underlying visual and non-visual processing around saccades, where occipital alpha power changes were related to the visually evoked responses [55]. Thus, the observed strong alpha power during natural reading may indicate words processing related visual-driven responses rather than oculomotor related non-visual-driven responses."

References:

55. Acunzo D, Melcher D. Changes in neural activity around the time of saccades: Separate

contributions of visual and non-visual signals on MEG alpha and theta band activity. PsyArXiv. 2021. doi:10.31234/OSF.IO/C29XM

Lines 149-155: I found the description of phase locking to saccade onset ("the main finding") and differences in phase locking as a function of lexical frequency (the most novel finding) hard to follow. It is not clear from the text and Figure 3 which comparisons are for phase locking to saccades towards the target item in general versus for high/low frequency targets. This should be made clear, as otherwise it is hard to interpret the main findings and the abstract and first sentences of the Discussion.

We thank the Reviewer for pointing this out and apologize for any confusion caused. We have now restructured Fig 3 and more details to clarify this (on page 12, line 213-227 with tracked changes):

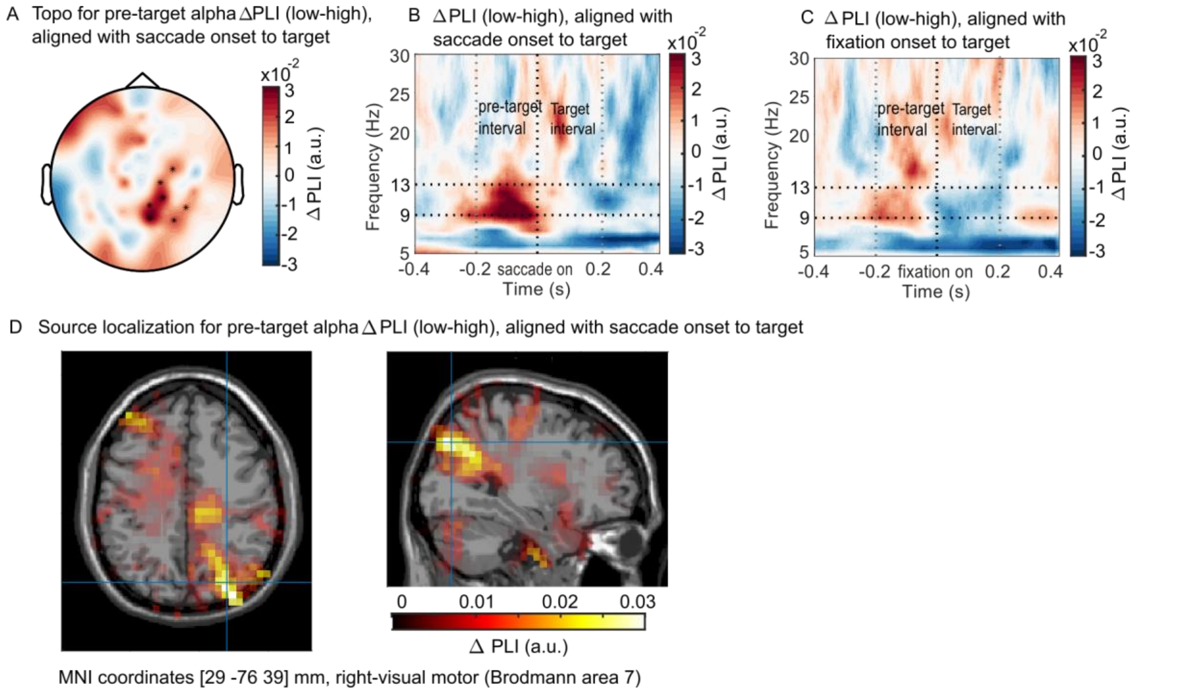

"Fig 3. Phase locking index (PLI) for the pre-target interval aligned with saccade onset to target words. *(A) Topography of the sensor clusters that showed a significant PLI difference in alpha band (9 – 13 Hz). The pre-target interval was aligned with the saccade onset towards target words (N = 38,* $P_{cluster} = 0.006$*, cluster-based permutation test). (B) The averaged PLI difference (*$PLI_{low} - PLI_{high}$*) over the significant sensor clusters in Fig 3A. (C) Using the same sensors as in Fig 3B but aligned with fixation onset towards the target, no robust effects were observed for the PLI difference during the pre-target interval. (D) Source localization of the alpha PLI difference during the pre-target interval in Fig 3B. The generator was localized to the right-visual motor cortex (Brodmann area 7) using an LCMV beamformer. Sacc., saccade."*

p. 161: From the text and figure it seems that only the 9-13 Hz band is considered, while higher and lower frequencies are not discussed. From a quick look at Figure 3, it seems possible that PLI might also have differed in these bands (see main comment above).

We thank the Reviewer for this comment, which prompted us to run the same phase locking index analysis for other frequency bands as well to get the full picture. Please see the response to the main comment above.

Figure 3B-D: missing "…set" in "saccade onset" (not clear what "saccade on" or "saccade on to target" means otherwise).

We thank the Reviewer for pointing out the inconsistency of terms used in the manuscript. We have now added 'set' in 'saccade onset' in Figure 3B-D (now is Figure 3A-C) as shown above in the updated manuscript.

Line 213: "This result indicates that the phase modulation originated from the visual cortex and was not biased by any eye movement related signals" - at best it "suggests" that the phase modulation was not biased by eye movement related signals. A good way to further support this idea would be to check if saccadic durations and magnitudes differed as a function of lexical frequency, since eye movement signals would tend to be correlated with the size and duration of the muscle movement.

We thank the Reviewer for this very important point, which prompted us to do two control analyses to make sure the PLI result is not biased by any eye movement related signals. We added this analysis as shown below (page 8, line 136-141 with tracked changes in the revised manuscript):

"In order to ensure that the phase modulation was not biased by any eye movement related signals, we compared the duration and amplitude of the saccades towards the target words with low and high lexical frequency. As shown in Fig 1D, no significant difference was observed for either saccade duration ($t_{(37)}$ = -0.77, P = 0.45, d = 0.13, two-tailed pairwise t-test) or saccade amplitude ($t_{(37)}$ = -1.35, P = 0.19, d = 0.22, two-tailed pairwise t-test)."

We added the result figure in the main text (on page 7, line 110-123 with tracked changes):

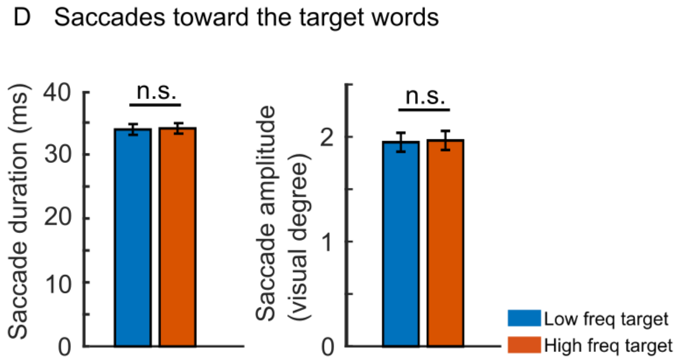

"Fig 1. (D) Duration (left panel) and amplitude (right panel) of the saccades towards the low and high frequency target words. n.s., not statistically significant."

Line 218 rewrite: "Also, this pre-saccadic modulation was purely phase-based that not driven by the power changes…"

We thank the Reviewer for this comment. We rewrote this sentence in the updated manuscripts on page 15 (line 289-293 with tracked changes):

"In addition, this pre-saccadic phase modulation was not driven by power changes, as suggested by the null finding of the pre-target alpha power difference between conditions (S6 Fig)."

Line 232: "This indicates that the previewed lexical information from the parafoveal word interacts with the processing of the foveal word." A pre-saccadic difference in phase alignment does not necessarily mean that the preview interacts with the foveal processing. It could be possible to check for such effects by looking at fixation-related potentials. This issue of parafoveal previews and post-saccadic effects should at least be considered.

We thank the Reviewer for pointing out this point and are sorry for the confusion. We agree with the Reviewer that the pre-saccadic phase alignment does not indicate an interaction between the parafoveal previews and post-saccadic processing. However, for 'interacts with the processing of the foveal word' we meant the processing of the pre-target word in the fovea rather than the post-saccadic processing of the target word. We rewrote this sentence in the updated manuscript on page 16 (line 311-314 with changes tracked):

"…… we did find that the consistency of the pre-saccadic alpha phase was modulated by the lexical frequency of the parafoveal word (Fig 3). This indicates that the lexical information from the parafovea affected the neural activity when processing the current words in the fovea."

Recent reviews:
Dimigen, O. & Ehinger, B.V. (2021). Regression-based analysis of combined EEG and eye-tracking data: Theory and Applications. Journal of Vision, 21, 3
Huber-Huber C, Buonocore A & Melcher D. (2021). The extrafoveal preview paradigm as a measure of predictive, active sampling in visual perception. Journal of Vision;21(7):12. doi: 10.1167/jov.21.7.12.

We thank the Reviewer for sharing these recently published reviews, which are highly relevant to our study and help us to have a better cover of the literature. We cited these two papers in the discussion section in the updated manuscript (on page 17, line 336-341 with tracked changes):

" Our results point to the importance of investigating visual processing in naturalistic scenarios rather than using a passive viewing paradigm. Now the methodological problems of doing so can be overcome [56] and naturalistic experiments have huge potential to reconceptualize the nature of human visual processing both on the level of behaviour and neural mechanisms [57]"

References:

56. Dimigen O, Ehinger B V. Regression-based analysis of combined EEG and eye-tracking data: Theory and applications. J Vis. 2021;21: 3–3. doi:10.1167/JOV.21.1.3

57. Huber-Huber C, Buonocore A, Melcher D. The extrafoveal preview paradigm as a measure of predictive, active sampling in visual perception. J Vis. 2021;21: 1–23. doi:10.1167/JOV.21.7.12

Lines 235-243: This seems to mainly be conjecture. You could easily eliminate it or it would need to be re-written to reflect the complicated relationship between attention shifts and saccade onset times that make it difficult to make any strong claims based on the effects presented in this ms.

We thank the Reviewer for this comment, which prompts us to rewrite this paragraph of "Alpha power gates attention during natural reading". We updated this in the new manuscript on page 16 (line 318-330 with tracked changes):

"It has been proposed that natural reading relies on the fast modulation of spatial visual attention, but how do these processes relate to neuronal oscillations? The hemispheric lateralization of alpha activity has been thought to index the deployment of visual attention [6,53]. In the scenario of reading sentences, visual attention shifts continuously through saccades, which was supported by the fact that the alpha activity remained strong during reading (Fig 2A). Similar alpha activity and hemispheric lateralization have been found in another natural reading task where eye movements were allowed [54]. Therefore, the stage is now set to further investigate how alpha activity during natural reading shapes the visual information flow in relation to hemisphere power modulation and saccades."

Lines 245-250: I see the point that the authors are trying to make, but this is tempered by the fact that saccade onset is a relatively well-defined moment and there is general agreement over how to measure it. Fixation onset is a completely different matter, with much less agreement over when a saccade is really complete. A simpler explanation is that saccadic onset estimates are more precise, in which case phase-locking estimates would benefit from this precision and errors in the hypothetical fixation onset would spread out these values and reduce phase locking. Indeed, even the authors spend some time in the Methods explaining their criterion for saccadic onset (lines 385-) but no details are given on how fixation onset was measured.

We thank the Reviewer for this comment, which we followed and helped us to explain our result better. We updated this information in the new manuscript on the following pages with tracked changes:

Discussion section (page 18, line 354-357): "Please note that the detection of saccade onsets is better defined and relatively easier than the detection of saccade offsets (i.e., fixation onsets). This could somehow bias the estimation of the phase-locking index to be stronger for saccade onsets than fixation onsets."

Method section (page 24, line 495-499): "A fixation onset (or saccade offset) was detected if all these parameters were below the thresholds and the pupil was not missing; the latter case was defined as

blinks. We disabled the fixation updates function by setting the fixation updates interval to zero so that the monitoring of the eye movements was as precise as the 1000 Hz sampling rate of the eye tracker."

I did not find the argument that estimates of saccade onset are more precise INTERNALLY (in the brain) than fixation onset compelling at all. It seems to me that using efference copy to internally predict saccade onset would be relatively imprecise while the timing of re-afferent visual input is much more precise.

We thank the Reviewer for this comment and agree with the Reviewer's opinion. We have deleted the argument about 'saccade onsets are more precise internally than fixation onsets', instead, we added the following text to the updated manuscripts on page 18 (line 347-349, with tracked changes):

"Since saccade initiation occurs before the visual stimuli land on our retina, the timing of neural activity in the visual cortex can be optimized to maximize the processing of the post-saccadic information."

Line 448: given that fixation durations in the condition of interest were matched, this is not a general problem, but it would be useful to have an estimate of how many of the -200 to 0 intervals contained a saccade.

We thank the Reviewer for this comment, which prompted us to run a new analysis to estimate the percentage of saccades during the -200 to 0 intervals. We added details about this analysis in the updated manuscript (on page 9, line 150-153, with tracked changes)

"This 0.2 s time window was the average duration for pre-target words as observed in Fig 1C, within which the percentage of saccades showed no significant difference between conditions ($t_{(37)}$ = 0.94, P = 0.35, pairwise t-test, two-sided)"

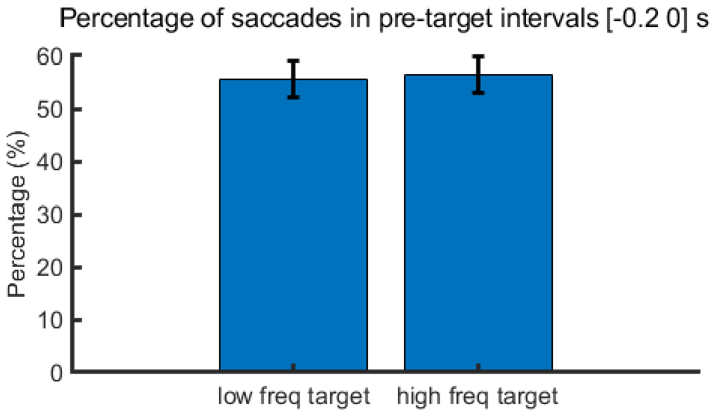

Fig R1. Percentage of saccades during the pre-target intervals (-0.2 to 0 s) under the conditions of low and high lexical target words. No significant difference was found for the percentage of saccades when the following target words were with low (left column) or high (right column) lexical frequency, as shown in Fig. R1 ($t_{(37)}$ = 0.94, P = 0.35, pairwise, two-sided t-test). Please note for each

participant, we made sure the number of pre-target intervals was the same across two conditions through a random selection procedure (for details please see Methods). Error bars indicate the standard error over participants (n = 38).

Reviewer #3: This study shows saccades aligned to alpha oscillations during natural reading, providing evidence for saccade timing controlled by these oscillations. This result is interesting as it extends the role of alpha oscillations as an organiser of brain activity in time to natural reading. I do not see any major issue with this work and believe that it will be well received by the community.

We thank the Reviewer for these positive comments and for the constructive questions that helped us further improve the manuscript.

I do have a few questions I hope the authors can comment on:
As the authors describe, evidence (from the same group and others) exists that saccades phase-lock to alpha oscillations in the visual system. I wonder whether the effect observed during natural reading is a specific case of this more general mechanism. In this way, the text read by the participants would be "just another" visual scene that is to be explored, and therefore dependent on alpha oscillations. Or do the authors think that natural reading is a distinct process and previous results from visual alpha oscillations do not necessarily hold?

We thank the Reviewer for this interesting comment, which prompted us to add more detail about the uniqueness of our study (on page 14, line 255-263 with tracked changes in the updated manuscript).

"A related effect has been found during free viewing of images: stronger alpha phase locking before saccades during exploration results in stronger memory encoding [22] (for a systematic review, see [40]). These findings are consistent with the notion that eye movement related signals modulate the ongoing oscillatory activity in the visual system to ensure better processing of the post-fixational stimuli [39]. In the current study, we extended this notion to the language domain by studying natural reading. Together, these findings from processing images and language suggest a general neural mechanism supporting the coordination between visual and oculomotor systems across different cognitive domains."

References:

22. Staudigl T, Hartl E, Noachtar S, Doeller CF, Jensen O. Saccades are phase-locked to alpha oscillations in the occipital and medial temporal lobe during successful memory encoding. PLoS Biol. 2017;15: 1–15. doi:10.1371/journal.pbio.2003404

39. Melloni L, Schwiedrzik CM, Rodriguez E, Singer W. (Micro)Saccades, corollary activity and cortical oscillations. Trends Cogn Sci. 2009;13: 239–245. doi:10.1016/j.tics.2009.03.007

40. Kragel JE, Voss JL. Looking for the neural basis of memory. Trends Cogn Sci. 2022;26: 53–65. doi:10.1016/J.TICS.2021.10.010

The current focus in language/speech research seems to be on theta oscillations. Their frequency would also match that of saccades during reading (~4 Hz), suggesting that theta might be ideal to organise language processing even during reading. Could the authors speculate on why alpha is still the important frequency here?

We thank the Reviewer for this comment as it helped us clarify the points that were not sufficiently clear in the original version of our manuscripts. We have now added a new paragraph in the discussion section to elaborate on this point (on page 15, line 294-301 with tracked changes in the revised manuscript):

"Although during natural reading, saccades occur in the frequency range of the theta band, we would like to stress that saccadic patterns are not oscillatory per se. Fixation times are determined by the properties of the fixated words such as lexical frequency. We have recently put forward a theoretical framework making explicit how alpha oscillations might serve to coordinate visual processing during visual exploration and reading [48]. Essentially the alpha oscillations serve to organize a pipelining mechanism operating to efficiently coordinate the different stages of word processing through the hierarchy in the ventral visual stream."

References:

48. Jensen O, Pan Y, Frisson S, Wang L. An oscillatory pipelining mechanism supporting previewing during visual exploration and reading. Trends Cogn Sci. 2021.

I also wonder whether results would be different if participants' actual comprehension had been tested.

We thank the Reviewer for pointing this out and are sorry for the confusion. In our current experimental design, 25% of the sentences were followed by a comprehension question. We have revised the illustration of the reading task in Fig 1A as shown below (updated in the new manuscript on the following pages with tracked changes).

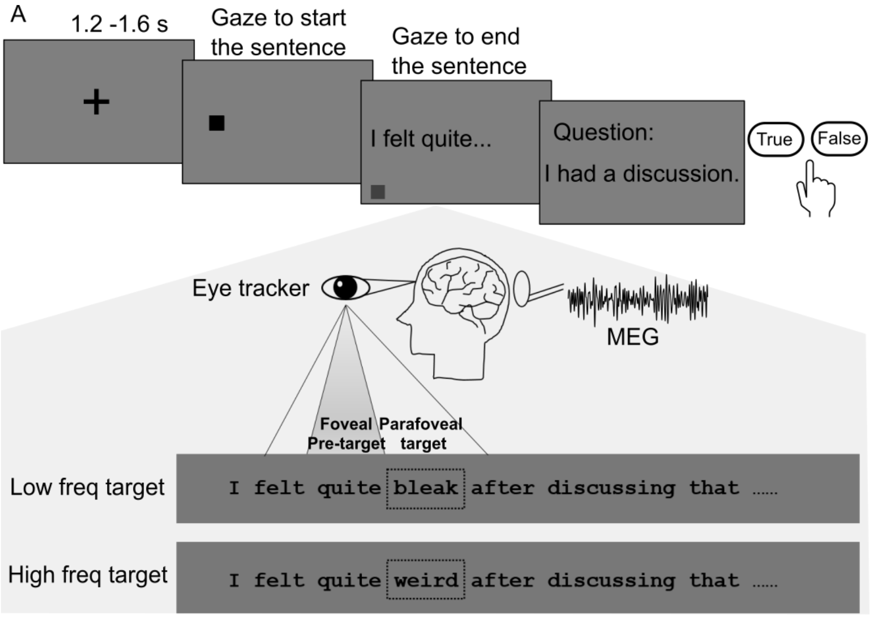

Main text (on page 7, line 110-116): "Fig 1. (A) Eye positions and brain activity were recorded simultaneously when participants (N = 38) read 228 one-line sentences. All sentences were plausible

with unpredicted target words of either low or high lexical frequency (denoted by the dashed rectangle; not shown in the experiment). One-quarter of the sentences were followed by a comprehension question about the just presented sentence to ensure careful reading."

Method section (on page 19, line 392-394): "Since all participants showed high accuracy for these questions (95.4% ± 4.7%, mean ± SD), which indicates a good comprehension of all the sentences, all trials were included in further analyses"

The significance of "target words" for participants was not completely clear to me, did they have to detect those words?

We thank the Reviewer for this comment, which we followed and helps us better present the paradigm. This is a natural reading task, and we did not check if participants detected the target words per se. The participants did need to answer comprehension questions by pressing a button in ¼ of the sentences. Given the high accuracy on comprehension (~95%) we do not think that target words were ignored. We have now revised the figure as shown in the above comment.

How does the power spectrum of a typical saccade look like - is it possible that the saccadic signal biases the analysis if smeared into the relevant time intervals (by the acausal filtering performed)?

We thank the Reviewer for pointing out this very important point. The power spectrum of a typical saccade is shown below (this figure is from Carl, C., Açık, A., König, P., Engel, A. K., & Hipp, J. F. (2012). The saccadic spike artifact in MEG. Neuroimage, 59(2), 1657-1667.). This paper showed that in MEG, the saccadic artefact is mainly in the gamma frequency band in the frontal and temporal regions.

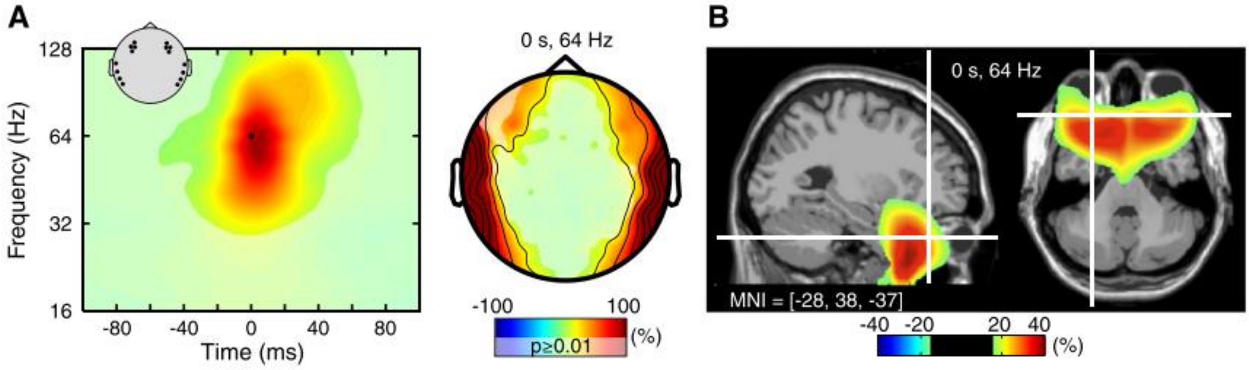

Fig. 4. Spectral characteristics of the SF. A. Power change at sensor level around the onset of saccades relative to baseline average from 400 ms to 350 ms before saccade onset. Unmasked regions denote significant difference to zero (*t*-test, p<0.01, FDR corrected). Left: power response resolved in time and frequency for selected regions of interest (indicated in the inset). Right: topography of the relative power changes for 64 Hz at saccade onset (time t = 0 ms). Unmasked regions denote significant difference from zero (*t*-test, p<0.01, FDR corrected). B. Sources for the sensor topography shown in A overlaid on the MNI template brain. Data are masked by statistics (*t*-test, p<0.01, FDR corrected).

We also performed two control analyses to make sure the PLI result is not biased by any eye movement related signals. We added this analysis as shown below (page 8, line 136-141 with tracked changes in the revised manuscript):

"In order to ensure that the phase modulation was not biased by any eye movement related signals, we compared the duration and amplitude of the saccades towards the target words with low and high lexical frequency. As shown in Fig 1D, no significant difference was observed for either saccade duration ($t_{(37)}$ = -0.77, P = 0.45, d = 0.13, two-tailed pairwise t-test) or saccade amplitude ($t_{(37)}$ = -1.35, P = 0.19, d = 0.22, two-tailed pairwise t-test)."

We added the result figure in the main text (on page 7, line 110-123 with tracked changes):

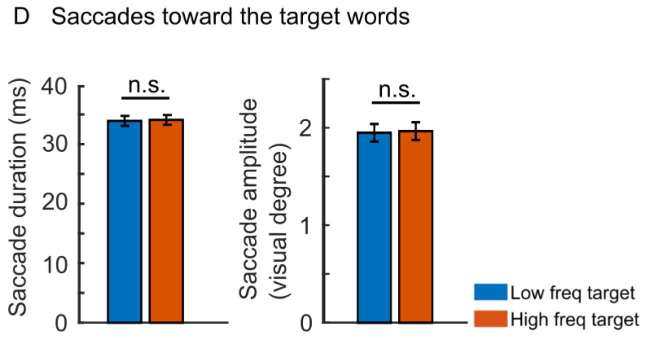

"Fig 1. (D) Duration (left panel) and amplitude (right panel) of the saccades towards the low and high frequency target words. n.s., not statistically significant."

Results and methods seem to focus on the condition difference, but I think results (Fig 3B,C) for overall phase-locking (not only the difference) would also be relevant. There is a peak in the alpha band for condition difference, is it also present for overall phase-locking when tested separately for the two conditions?

We thank the Reviewer for this point, which helped us to clarify our analyses. We now show the same phase locking index analysis for the low and high lexical target conditions separated, as shown below (on the following pages in the revised manuscript with tracked changes):

Result section (on page 10, line 186): "…… for the raw PLI for each condition please see S3 Fig"

Supplementary material (on page 39):

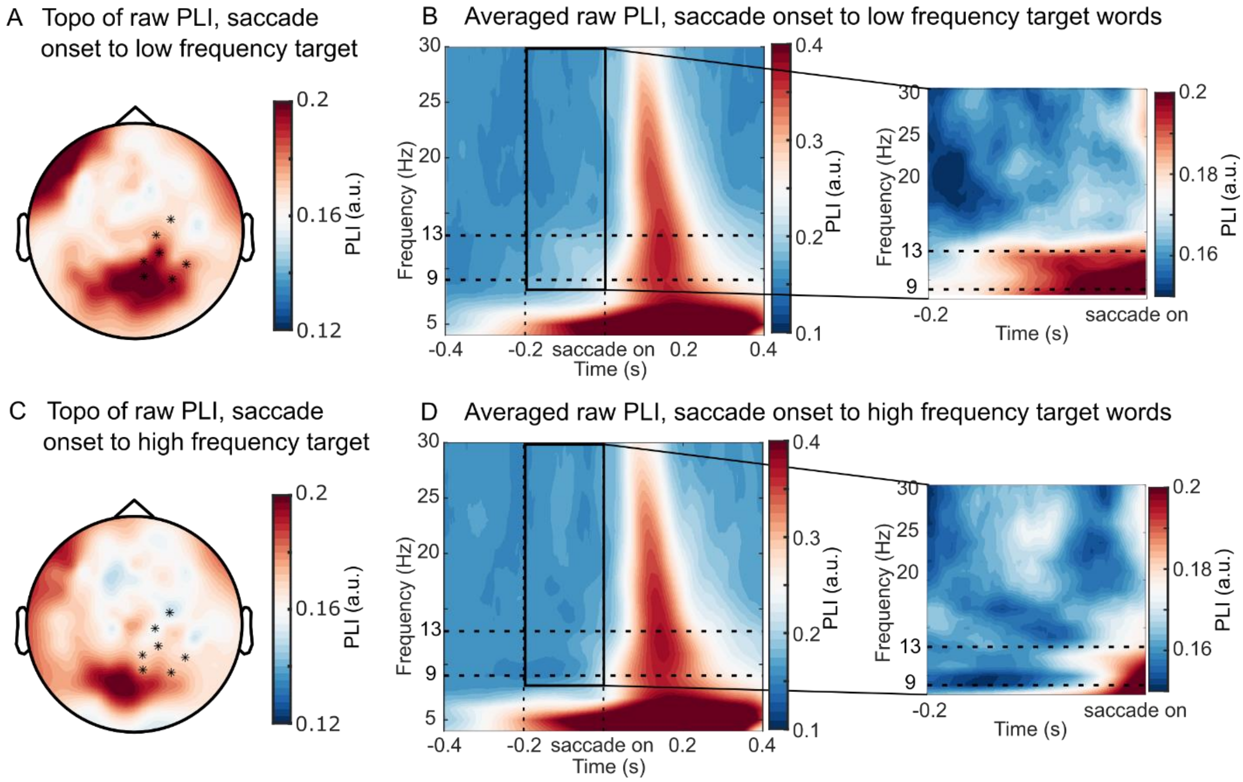

"S3 Fig. Raw phase locking index (PLI) for the pre-target interval aligned to saccade onset to the low and high lexical target words. (A) Topography of the phase locking index with respect to low lexical target words averaged over the pre-target interval (-0.2 to 0 s) and the alpha frequency band (9-13 Hz). The black stars indicate the sensor from the cluster in Fig 3(B). (B) Averaged PLI over the sensors in the cluster aligned with the onset of the saccade (zero time point) to the low lexical target words. The graph on the right is a zoom-in on the PLI from -0.2 to 0 s, 8 to 30 Hz. (C) and (D) are the same as (A) and (B) but for the condition of high lexical target words."
