# Supplementary material for: Saccades are locked to the phase of alpha oscillations during natural reading
Source: PLoS Biol. 2023 Jan 17;21(1):e3001968. doi: 10.1371/journal.pbio.3001968 (PMC9882905; doi:10.1371/journal.pbio.3001968)
Supplement: Attachment — Submitted filename: Responses to editorial requests.pdf [file pbio.3001968.s012.pdf]

Editorial requests :

Thank you for your patience while we considered your revised manuscript "Saccades are locked to the phase of alpha oscillations during natural reading" for publication as a Short Reports at PLOS Biology. This revised version of your manuscript has been evaluated by the PLOS Biology editors, the Academic Editor and the original reviewers 1 and 3. Reviewer 2 was not available to re-review.

Based on the reviewers comments and our discussion with both the reviewer and our Academic Editor, we are likely to accept this manuscript for publication. As you will see from their comments below, the two reviewers were rather split in their views in this round. Reviewer 1 continued to raise some questions about the alpha phase differences, though during some back-and-forth discussion, Reviewer 1 did acknowledge that that one might not expect consist phase differences between conditions if phase locking is weaker for low-frequency words. However, Reviewer 1 still questioned how strongly the study can inform on our mechanistic understanding alpha and inhibition during reading absent information about specific phases. Given that our readership might have similar questions and concerns to those still being expressed by Reviewer 1, we ask that you explicitly comment on these points in the final version of the article.

We thank the Editor very much for the likely acceptance of our manuscript and for the insightful suggestions that helped us further improve it. We added some comments on this point in the final version of the article (Page 8 with tracked changes), which are copied as below:

"Although the alpha phase before making saccades is more consistent over trials when previewing a low frequency compared to a high frequency word, the actual preferred alpha phase in each condition is not consistent across participants (S4 Fig, B). This is likely because the underlying cortical dipolar generators of the alpha activity have different orientations with respect to the MEG sensors across participants. The variability in dipole orientation results in different absolute phases of the alpha oscillations in the MEG sensors. Also, we did not find a systematic difference in absolute preferred phase when comparing saccades towards low- and high frequency words (see S4 Fig, F). We conclude that the strength of alpha phase locking differed between conditions but not the actual preferred phase. This absence of phase-difference is consistent with a mechanism in which saccades typically are locked to the same phase of the alpha oscillations, but this locking can be stronger or weaker. Future studies based on intracranial recordings in human or non-human primates would allow for estimating the absolute oscillatory phase to which saccades are locked."

Please also make sure to address the data and other policy-related requests listed below my signature are fully addressed. Addressing these issues is necessary for publication and incomplete responses and edits will result in publication delays.

We thank the Editor for pointing out the data sharing issue. We added an excel file (S1_Data) to store all the data needed to reproduce the figures in the main text and the supplementary information.

As you address these items, please take this last chance to review your reference list to ensure that it is complete and correct. If you have cited papers that have been retracted, please include the rationale for doing so in the manuscript text, or remove these references and replace them with relevant current references. Any changes to the reference list should be mentioned in the cover letter that accompanies your revised manuscript.

We confirm that no changes have been made to the reference list.

Reviewer #1:

I appreciate the attention brought by the authors to the comments and suggestions I provided after reviewing the first version of their manuscript.

Although the discussion of the results is now more in line with the relatively modest effects reported, I still see issues with the significance of the effects reported, their interpretation in terms of neurophysiological mechanisms, and with the methodological approach to source imaging.

We thank the Reviewer for these comments, and hope our updated version of manuscript will address the issues.

In terms of the significance of the effects, I am grateful for the efforts put in testing the consistency of the phase differences observed between the experimental conditions and across participants. It is indeed disappointing that the actual values of the alpha phase are not systematic between participants, and to a more substantial concern, that their differences between conditions within participants are not consistent. This latter is concerning because, although the absolute phase of the alpha oscillations can be difficult to detect and interpret across participants, as the authors now explained, the relative difference of the phase values should be interpretable. Indeed, if the alpha cycle marks the phases of relative greater/lesser inhibition, the phase differences between conditions should be expected to indicate a systematic change at least towards the direction of a trough or a peak of the oscillation, across participants. Without this result, I find the report less convincing and still quite far from a mechanistic and generalizable interpretation of the findings.

We thank the Reviewer for this comment, we have added some cautions about how to explain our results in the updated version of the manuscript, please see the response to the editor above. In particular we point to the need for intracranial recordings in humans and animals to resolve the issue of absolute phase. We expect that our study will inspire such investigations.

The explanation provided concerning the LCMV weights applied to a complex signal in lieu of the physical MEG physical measures is not convincing either unfortunately. The authors cite a 2008 paper that used a similar approach, and a Fieldtrip email thread where the argument is that "it should basically work." I don't believe this is convincing.

We thank the Reviewer for pointing out the validity of the source localization method that we used in the study. However, we would like to argue that the method is based on a published paper in Neuroimage and has been suggested by the FieldTrip team. Based on the Neuroimage paper we believe that our LCMV findings are reliable; however, we would like to stress that the core conclusion of our study is based on the sensor level data.

The source maps have been recomputed using a longer time segment for the computation of data covariance (thank you). For completeness, the display of Figure 3D should show MR crossections in all three directions (axial, coronal and sagittal) and using a glass-brain rendering so that the reader can better appreciate the location and extension of the activated regions.

We thank the Reviewer for this helpful suggestion, which we followed. We now show the source results from all three directions in the updated version of the article (Page 10 with tracked changes), which are copied as below. We did not include the glass brain as it will provide redundant information.

D Source localization for ΔPLI (low-high) in alpha band during the pre-target intervals, aligned with saccade onset to the target words

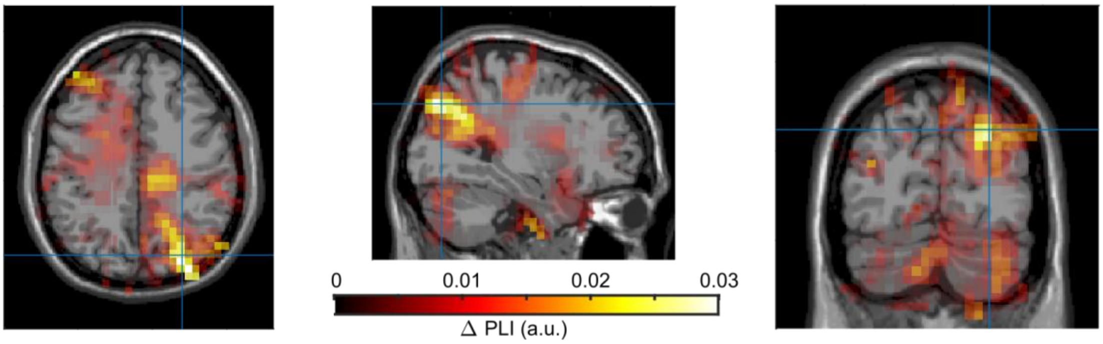

MNI coordinates [29 -76 39] mm, the right-visual motor (Brodmann area 7)

In general, all delta_PLI effects should be reported and plotted as relative values i.e, in percentages of PLI statistics changes between conditions. It would make clearer that the effects reported are remarkably small, and based on a relatively small cohort.

We thank the Reviewer for this point, however, the PLI values are bound between 0 and 1. In this case, deriving relative difference values is problematic as we might divide by close-to-zero values (as thus contributing to – rather than taming – the variance. As such we think it is better to apply the difference PLI in the alpha band (Fig 3 in the main text) and delta band to make sure a fair comparison. We believe this is justified as the PLI measure is bound between 0 and 1 and thus well-behaved; certainly, our procedure will not create spurious results.

Minor:

p.8 - "The alpha phase during the pre-target interval was significantly stronger when previewing words with lower lexical frequency in the parafovea...": the alpha phase cannot be stronger. I believe this is a typo and that the authors refer to alpha phase locking.

Thanks, now resolved.
